# Supplementary material for: Bird populations most exposed to climate change are less sensitive to climatic variation
Source: Nat Commun. 2022 Apr 19;13:2112. doi: 10.1038/s41467-022-29635-4 (PMC9018789; doi:10.1038/s41467-022-29635-4)
Supplement: Supplementary file 1 — Supplementary Information [file 41467_2022_29635_MOESM1_ESM.pdf]

Supplementary Methods

Impact of using non-gridded temperature data

Our analysis in this manuscript predominantly relied on the temperature data from the E-OBS Gridded Dataset v17.0 with a resolution of 0.25 degrees[1]; however, at two sites (Vlieland, Netherlands and Sicily, Italy) gridded data were not available during the study period and alternative sources of temperature data were required. For Vlieland, we interpolated daily mean temperature information from weather stations provided by the Royal Dutch Meteorological Institute (KNMI) on the nearby island of Terschelling (<33km away). For Sicily, temperature data were taken from weather stations operated at the study site by a co-author (C. Cusimano). Using a combination of gridded temperature data and local weather stations can be problematic as each dataset may have different biases and uncertainties that can affect the results of our analyses. To ensure that the use of local weather station data does not influence our result we re-fitted models with data from Vlieland and Sicily excluded. Tables with model coefficients from this supplementary analysis are shown below. Our conclusions were unaffected by the removal of these two study sites (Supplementary Table 6-7).

Modelling spatial autocorrelation in climate change sensitivity

To account for spatial autocorrelation in phenological sensitivity we included a Matérn correlation function as a random intercept term in our model of phenological sensitivity. The Matérn correlation function between two points with Euclidian distance (d) greater than 0 is defined in equation 1. With d in units of degrees. K_ν is the modified Bessel function of the third kind [2].

$$2^{(1-\nu)}(\rho\ d)^{\nu}\frac{\mathrm{K}_\nu(\rho\ d)}{\Gamma(\nu)} \qquad (1)$$

This function is optimized to estimate ν, smoothness parameter, and ρ, range parameter.

The model estimates a Matérn correlation function with a smoothness parameter (ν) of 0.218 and range parameter (ρ) of 0.589 (Supplementary Figure 5). Estimated pairwise correlation from this function is included in our analysis of phenological sensitivity to account for spatial autocorrelation between sites. To visualise patterns of phenological sensitivity accounting for spatial autocorrelation we predicted phenological sensitivity with interpolation to fill in non-observed locations. We see a latitudinal gradient in sensitivity, with more northerly latitudes showing higher sensitivity, particularly within the UK, and lower sensitivity in more easterly populations (Supplementary Figure 6).

For more details on the Matérn function and interpolation used for plotting see the help documentation of the spaMM package in R[3].

Explanation on how a covariance between the exposure and sensitivity affects the mean and variation in expected phenological advancement

We found a negative covariance in the exposure and sensitivity of great and blue tit populations: the populations with the strongest exposure tended to be the least sensitive. Intuitively one might expect that a negative covariance will reduce intra-specific variation in expected phenological advancement compared to a species in which there is no covariance between the sensitivity and exposure, while a positive covariance will increase the intra-specific variation. Here we explore this intuition formally, by mathematically describing how any among-population covariance between sensitivity and exposure will affect the mean and variance in expected phenological advancement for the entire species.

We will compare two species that have the same mean and variance in sensitivity (X) and exposure (Y) but where one species has a negative covariance between sensitivity and exposure ($\mathrm{COV}(X, Y) < 0$), while the other species shows no covariance between sensitivity and exposure ($\mathrm{COV}(X, Y) = 0$). We are interested in how $\mathrm{COV}(X, Y)$ affects the mean (expectation) and variance in expected phenological advancement (XY, the product of X and Y) for the species as a whole.

How covariance between sensitivity and exposure affects the mean expected phenological advancement

Probability theory tells us that the expectation (E) of the product of two independent variables X and Y is given by:

$$\mathrm{E}[X * Y]_i = \mathrm{E}(X) * \mathrm{E}(Y) \quad (2)$$

Probability theory also tells us that the expectation of the product of two dependent variables is given by:

$$\mathrm{E}[X * Y]_d = \mathrm{E}(X) * \mathrm{E}(Y) + \mathrm{COV}(X, Y) \quad (3)$$

From this it can be seen that a negative covariance lowers the expected value of a species mean phenological advancement if we compare it to a species in which sensitivity X and exposure Y are independent, since:

$$\mathrm{E}[X * Y]_d - \mathrm{E}[X * Y]_i = \mathrm{COV}(X, Y) \quad (4)$$

Furthermore, the relative contribution of any covariance between sensitivity and exposure to the observed expected phenological advancement ($R_{c \to \mathrm{E}[X*Y]_d}$) can be quantified as:

$$R_{c \to \mathrm{E}[X*Y]_d} = \frac{\mathrm{E}[X * Y]_d - \mathrm{E}[X * Y]_i}{\mathrm{E}[X * Y]_d} = \frac{\mathrm{COV}(X, Y)}{\mathrm{E}(X) * \mathrm{E}(Y) + \mathrm{COV}(X, Y)} \quad (5)$$

In our dataset we found that mean exposure is $\mathrm{E}(X) = 0.0354$ ˚C/year, mean sensitivity is $\mathrm{E}(Y) = 3.614$ days advancement/˚C and the covariance $\mathrm{COV}(X, Y) = 0.00361$. This means that $R_{c \to \mathrm{E}[X*Y]_d} = -0.029$ which tells us that the covariance between sensitivity and exposure lowered the mean expected phenological advancement by only 2.9% compared to a situation with no covariance.

Although we have shown above that the mean expected phenological advancement across all populations was little affected by the covariance, we emphasize that for calculating the expected advancement of a single population with a given sensitivity it can make a huge difference whether one uses the population specific exposure or the exposure averaged across all populations.

How covariance between sensitivity and exposure affects the intraspecific variation in expected phenological advancement

Probability theory tells us that the variance (V) of the product of two independent variables is given by:

$$\mathrm{V}[X*Y]_i = \mathrm{V}(X)*\mathrm{V}(Y)+\mathrm{V}(X)*\mathrm{E}(Y)^2+\mathrm{V}(Y)*\mathrm{E}(X)^2 \qquad (6)$$

Probability theory also tells us that the variance (V) of the product of two dependent variables is given by:

$$\mathrm{V}[X*Y]_d = \mathrm{V}(X)*\mathrm{V}(Y)+\mathrm{V}(X)*\mathrm{E}(Y)^2+\mathrm{V}(Y)*\mathrm{E}(X)^2 \qquad (7)$$
$$+\mathrm{E}(X)^2*\mathrm{E}(Y)^2+\mathrm{COV}(X^2,Y^2)-(\mathrm{COV}(X,Y)+\mathrm{E}(X)*\mathrm{E}(Y))^2$$

The influence of a negative covariance on the intraspecific variance of a species expected phenological advancement can thus be quantified as:

$$\mathrm{V}[X*Y]_d-\mathrm{V}[X*Y]_i = \mathrm{E}(X)^2*\mathrm{E}(Y)^2+\mathrm{COV}(X^2,Y^2)-(\mathrm{COV}(X,Y)+\mathrm{E}(X)*\mathrm{E}(Y))^2 \qquad (8)$$

From this it can be seen that the contribution of any covariance to the intraspecific variance is more complex, as it also depends on other factors (i.e. on $\mathrm{COV}(X^2,Y^2)$, $\mathrm{E}(X)$ and $\mathrm{E}(Y)$). Consequently, a negative covariance does not in all cases reduce the intra-specific variability in phenological advancement.

Finally, the relative contribution of any covariance between sensitivity and exposure to the observed variance in phenological advancement (R_c) can be quantified as:

$$R_{c\rightarrow V[X*Y]_d} = \frac{\mathrm{V}[X*Y]_d - \mathrm{V}[X*Y]_i}{\mathrm{V}[X*Y]_d} = \tag{9}$$

$$\frac{\mathrm{E}(X)^2 * \mathrm{E}(Y)^2 + \mathrm{COV}(X^2, Y^2) - \left(\mathrm{COV}(X,Y) + \mathrm{E}(X) * \mathrm{E}(Y)\right)^2}{\mathrm{V}(X) * \mathrm{V}(Y) + \mathrm{V}(X) * \mathrm{E}(Y)^2 + \mathrm{V}(Y) * \mathrm{E}(X)^2 + \mathrm{E}(X)^2 * \mathrm{E}(Y)^2 + \mathrm{COV}(X^2, Y^2) - \left(\mathrm{COV}(X,Y) + \mathrm{E}(X) * \mathrm{E}(Y)\right)^2}$$

In our dataset we found that variance in exposure is $\mathrm{V}(X) = 0.00749$ (˚C/year)^2, variance in sensitivity is $\mathrm{V}(Y) = 1.1867$ (days advancement/˚C)^2 and the covariance $\mathrm{COV}(X^2, Y^2) = -0.00332$. As a result $R_{c\rightarrow \mathrm{V}[X*Y]_d} = -0.2367$ and thus the covariance lowered the intraspecific variance in phenological advancement by 23.7% compared to a situation in which there was no covariance.

Simulated correlation between phenological sensitivity and climate change exposure

Our results showed a negative correlation between the exposure and sensitivity of great and blue tit populations. Populations with the strongest exposure tended to be the least sensitive to temperature change. Although this result is interesting, a concern could be that such a correlation may arise due to a mathematical dependency between exposure (temperature/time) and sensitivity (laying date/temperature) as both variables are dependent on the same temperature variable.

Specifically, populations may show different levels of inter-annual temperature variation, such that some populations show large temperature variation year to year while others show little. Large inter-annual temperature variation may mask any long-term trends in temperature over time, thus reducing our ability to accurately estimate exposure. Conversely, large inter-annual temperature variation may enhance our ability to estimate patterns of sensitivity as we are able to observe phenological responses across a wide range of environmental temperatures. In combination, this may lead to a negative correlation between exposure and sensitivity simply because both variables are derived from temperature. Those populations experiencing large inter-annual temperature variation will be those exhibiting higher sensitivity and lower exposure.

To understand whether such a relationship could explain our results we simulated the correlation between the exposure and sensitivity for data where no relationship exists between the two variables. We considered three different scenarios: small, medium, and large differences in inter-annual temperature variation between populations, with 1,000 iterations run in each scenario. Each iteration returned the Pearson's correlation between exposure and sensitivity from 500 populations each with 30 years of data (the mean study length in our observed data).

In the simulation, each population (i) has an exposure (E_i) and sensitivity (I_i), defined in equations 10 and 11 below.

$$E_i = E_{obsv} + \hat{E} \tag{10}$$

Where $\hat{E}$ is drawn from a normal distribution $\mathcal{N}(0, E_\sigma)$ and E_{obsv} and E_σ represent, respectively, the observed mean and standard deviation of climate change exposure in our study populations.

$$I_i = I_{obsv} + \hat{I} \tag{11}$$

Where $\hat{I}$ is drawn from a normal distribution $\mathcal{N}(0, I_\sigma)$ and I_{obsv} and I_σ represent, respectively, the observed mean and standard deviation of phenological sensitivity in our study populations.

Temperature in year t is then defined as:

$$temp_t = t * E_i + \varepsilon_{temp} \tag{12}$$

Where ε_{temp} is drawn from a normal distribution $\mathcal{N}(0, \sigma_{temp} + T_i)$, with σ_{temp} being the residual deviance in exposure observed in our study populations and T_i represents the added simulated effect of inter-annual temperature variation in population i. In this way a simulated population with no inter-annual temperature variation will exhibit the same level of residual deviance as our study populations. T_i is itself draw from a normal distribution $\mathcal{N}(0, x)$ where x is

used to define our three simulation scenarios small, medium, and large differences in inter-annual temperature variation between populations. $\sigma_{temp} + T_i$ is restricted to be no smaller than 0 to prevent negative standard deviation in residual deviance.

Finally, laying date in year t is defined as:

$$LD_t = temp_t * I_i + \varepsilon_{LD} \tag{13}$$

Where ε_{LD} is drawn from a normal distribution $\mathcal{N}(0, \sigma_{LD})$, with σ_{LD} being the residual deviance in sensitivity observed in our study populations.

With equations 10 to 13 we can simulate values of temperature and laying date for all years and populations. As a final step, we then fit two general linear models for each population to estimate sensitivity and exposure in our simulated data and calculate the Pearson's correlation coefficient between these variables. In this way we can simulate how inter-annual variation in temperature may affect our ability to estimate sensitivity and exposure and potentially drive a correlation between these variables where none exists. We simulated correlations for values of x at 0.1, 0.2, and 0.3 to represent small, medium, and large differences in inter-annual temperature variation between populations.

We found no evidence of correlation between exposure and sensitivity in our simulations regardless of the amount of inter-annual variation in temperature. The mean Pearson's correlation across 1,000 iterations was close to 0 in all cases ($6.00e^{-4}$, $1.73e^{-3}$, and $-2.73e^{-3}$ for values of x 0.1, 0.2, and 0.3 respectively; Supplementary Fig. 7). These simulation results show that a negative correlation

between exposure and sensitivity, as observed in our analysis, cannot be explained by differences in inter-annual temperature variation between populations.

Supplementary Figures

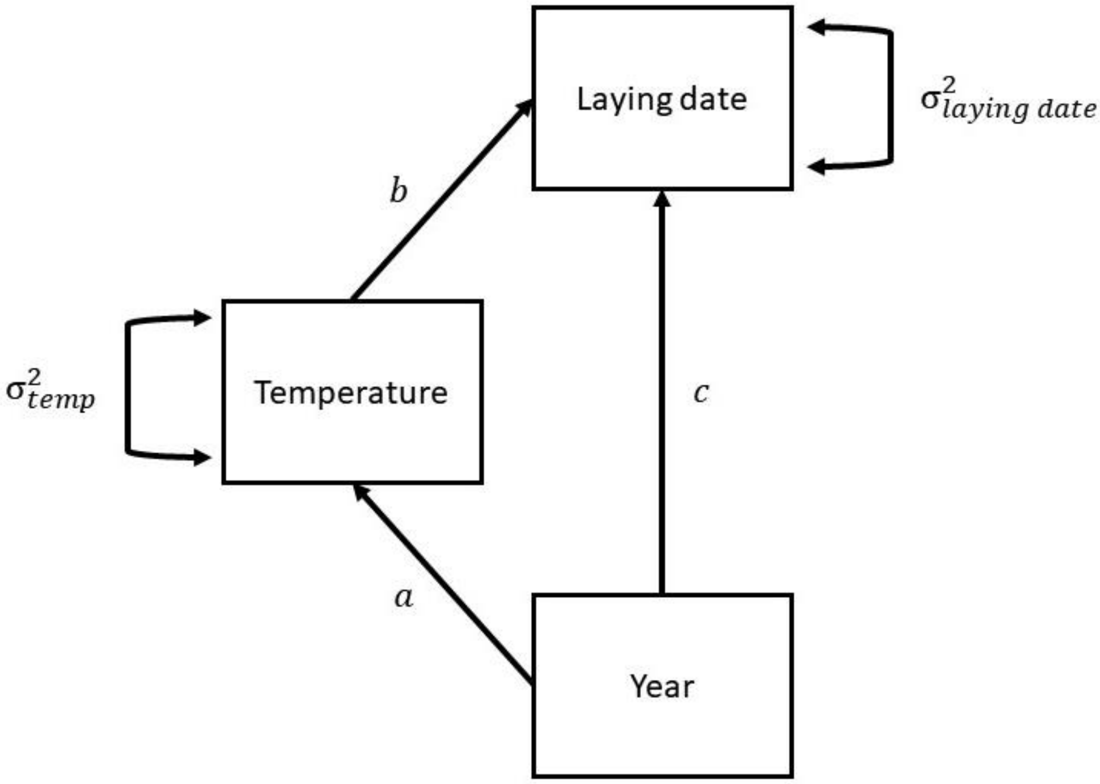

Supplementary Figure 1: Path diagram of the structural equation model used to account for trends in laying date over time that are not caused by temperature. The effect of time (Year) on laying date is separated into both a direct and indirect pathway. The indirect pathway accounts for effects of time that occur via changes in temperature, such as climate change, which is the product of climate change exposure (a) and phenological sensitivity (b). The direct pathway accounts for any changes in laying date over time that occurred through effects other than temperature change (c). $\sigma^2_{laying\ date}$ and σ^2_{temp} represent variance in laying date and temperature respectively. The Temperature variable used in the model of each population was derived from population-specific temperature windows.

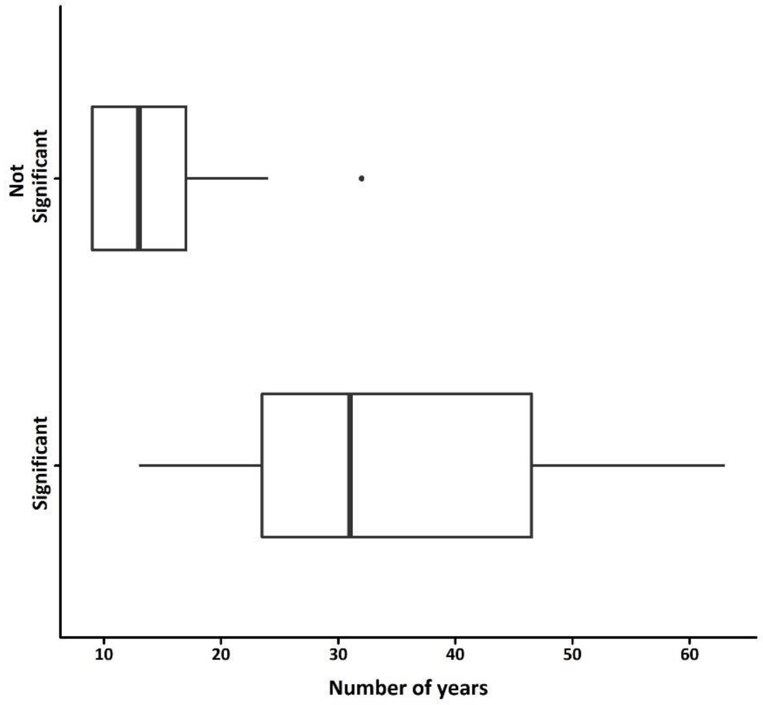

Supplementary Figure 2: Relationship between population sample size (number of years) and temperature window detection. Temperature windows were identified using a cut-off of $P_{AICc} \leq 0.05$. Those populations with temperature windows ≤ 14 days were excluded. Populations with no clear temperature window (n = 20 biologically independent populations) had fewer years of data than those where a temperature window was detected (n = 47 biologically independent populations). Temperature windows will be more difficult to identify in datasets with limited sample size [4]. Those cases without a clear temperature window are therefore likely a consequence of limited statistical power. Boxplots shows median (centre line), 25th and 75th quantiles (lower and upper hinges), and 1.5x inter-quartile range (whiskers). Observations outside further than 1.5x inter-quartile range are shown as outlier points.

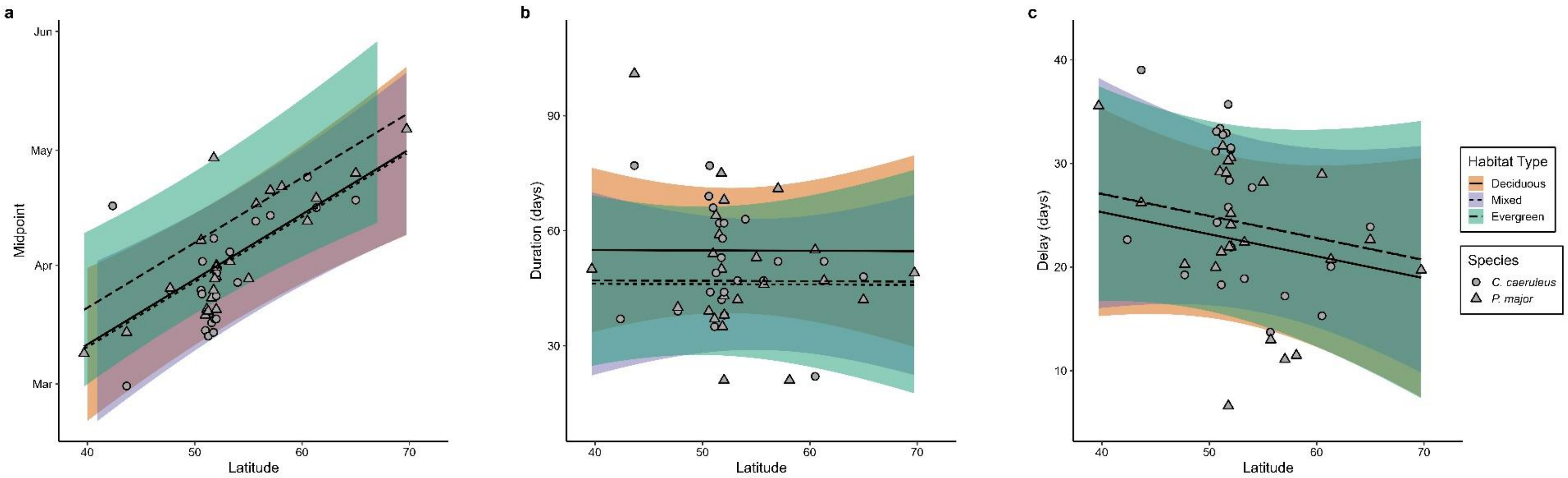

Supplementary Figure 3: Relationship between latitude and temperature window characteristics. Change in temperature window a) midpoint, b) duration, and c) delay between midpoint and mean annual laying date in deciduous (orange), mixed (blue), and evergreen (green) habitats. There is a clear relationship between latitude and temperature window midpoint. Temperature window duration and delay are unaffected. Plots show model predictions and 95% prediction intervals.

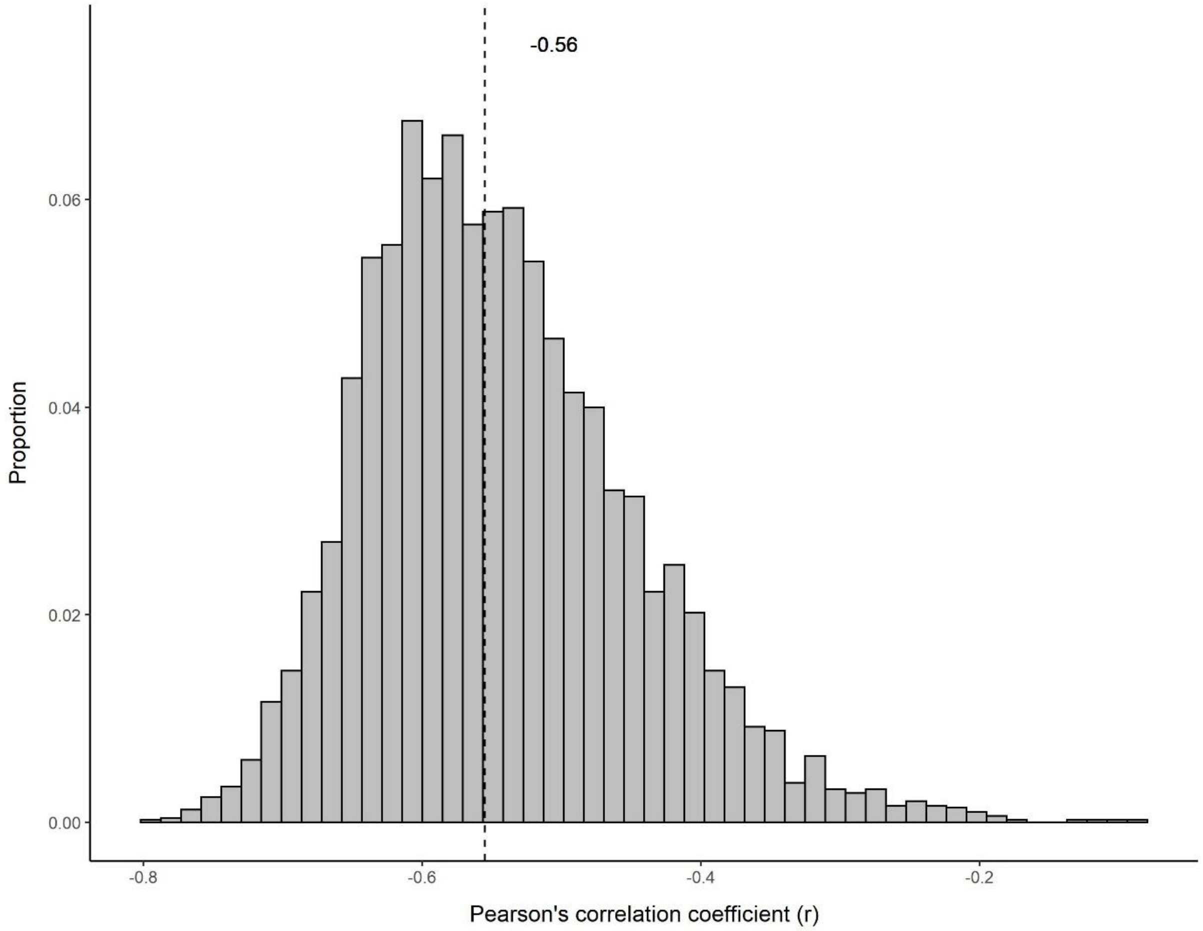

Supplementary Figure 4: Distribution of Pearson's correlation coefficients between phenological sensitivity and climate change exposure following non-parametric bootstrapping. Correlation coefficients generated from ordinary non-parametric bootstrapping with 5,000 iterations. Median correlation coefficient represented by the dashed vertical line (*r* = 0.56).

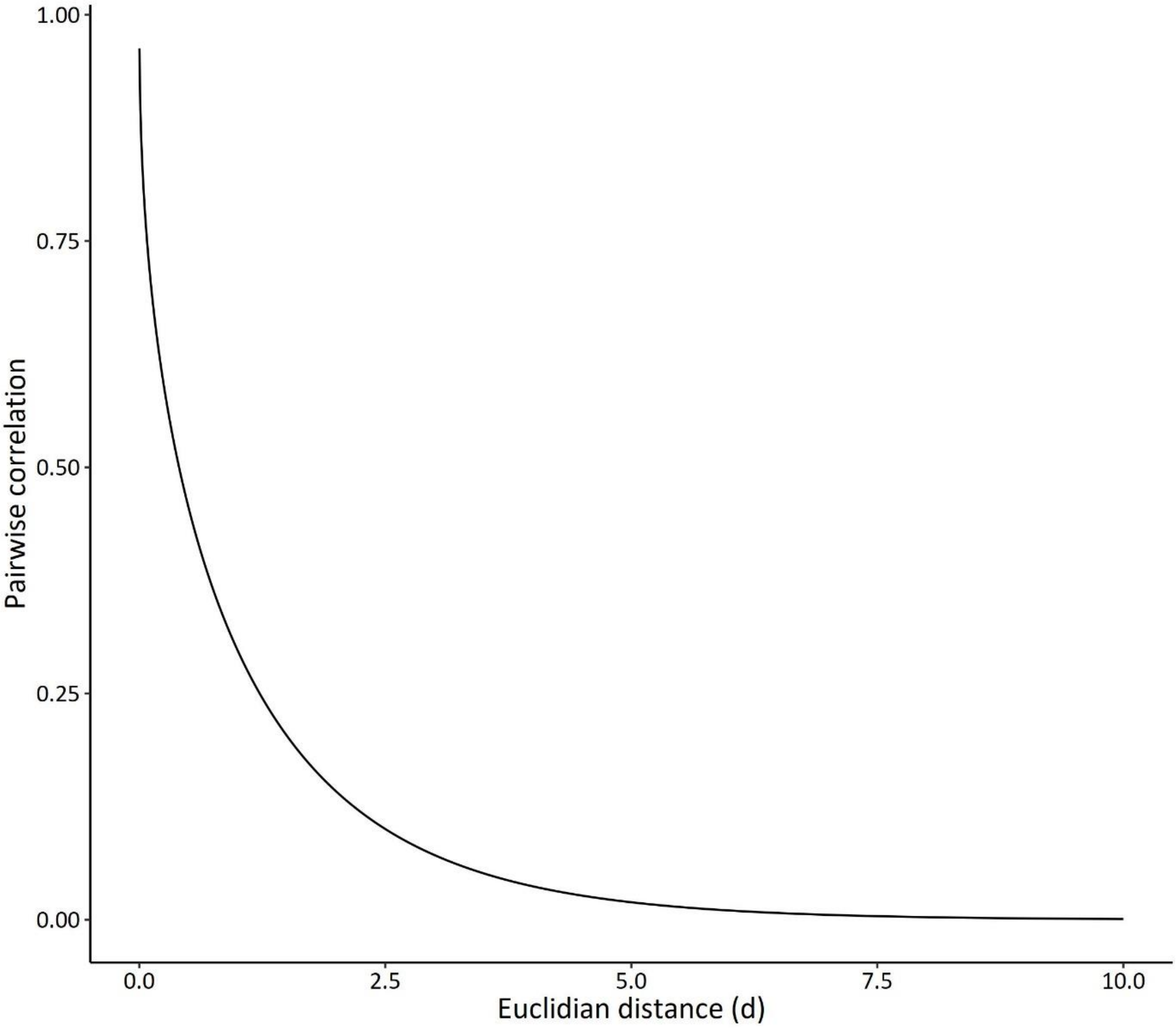

Supplementary Figure 5: Estimated relationship between Euclidian distance (d) in degrees and pairwise correlation from our fitted Matérn correlation function ($\nu = 0.218;\ \rho = 0.589$).

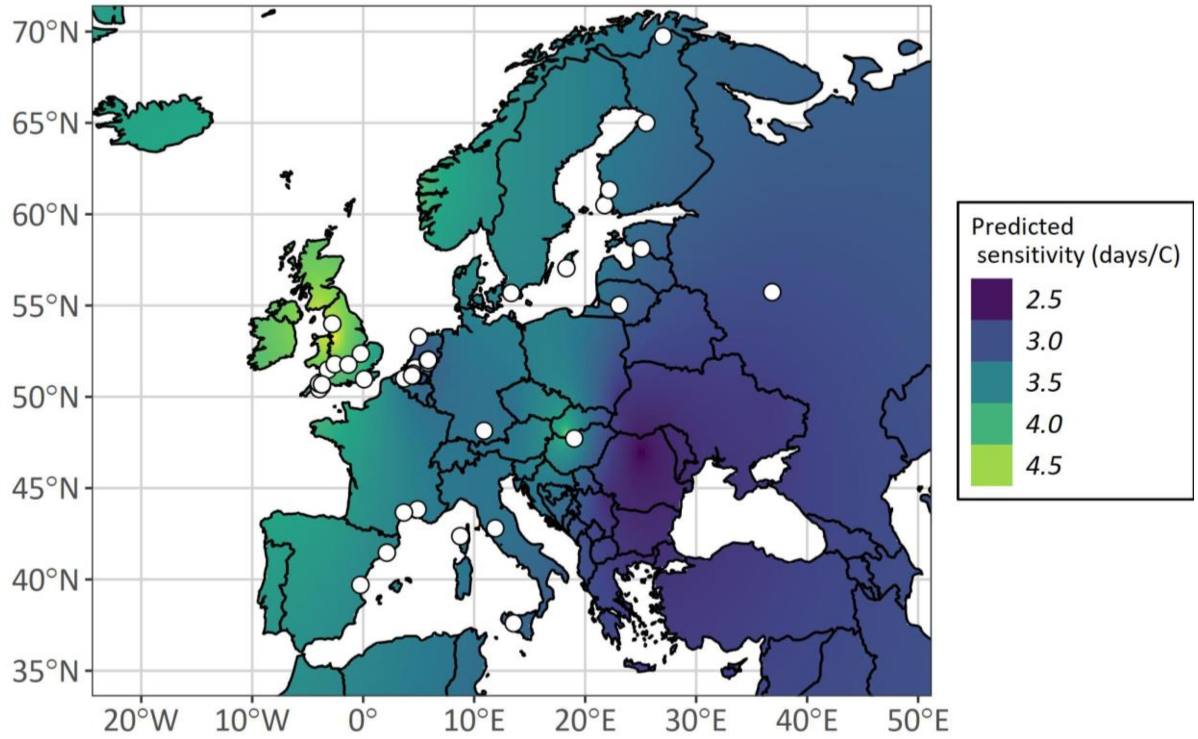

Supplementary Figure 6: Interpolated phenological sensitivity accounting for spatial auto-correlation from our fitted Matérn correlation function ($\nu = 0.218;\ \rho = 0.589$). White points show study population locations. Dark blue represents areas with low predicted sensitivity. Lighter green represents areas with higher predicted sensitivity.

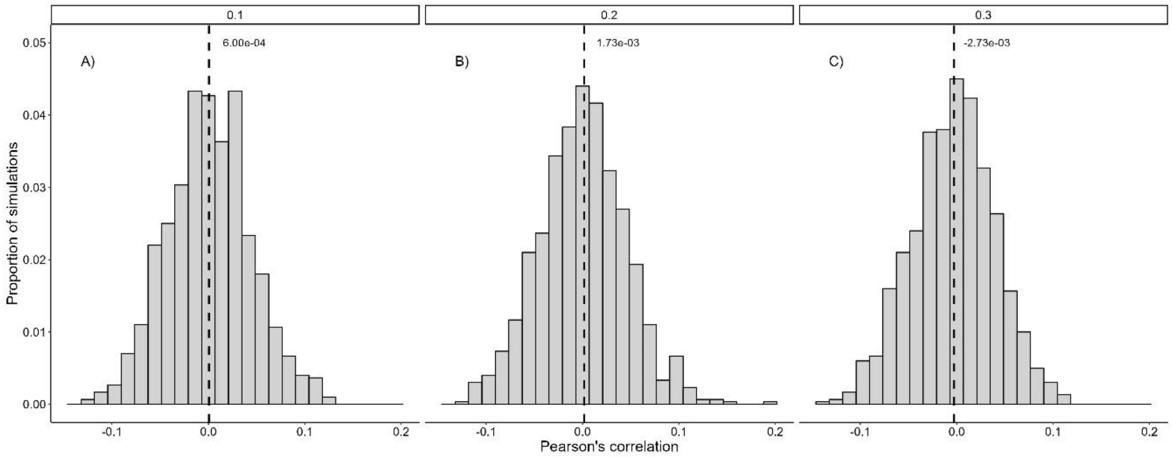

Supplementary Figure 7: Histogram of simulated correlations between climate change exposure and phenological sensitivity. Panels show simulations with A) small, B) medium, and C) large differences in inter-annual temperature variation between populations. In all cases the median correlation from 1,000 simulations was close to 0.

Supplementary Tables

Supplementary Table 1: Likelihood-ratio test results comparing models with and without species interactions. Coefficient estimates of all models with interactions are provided in Supplementary Table 4 and 5.

Response variable	Log-likelihood (interaction model)	Log-likelihood (non-interaction model)	χ2	Degrees of freedom	p-value
Midpoint	-163	-166	5.88	2	0.053
Duration	-190	-191	1.99	2	0.370
Delay	-151	-148	5.10	2	0.078
Phenological sensitivity	-43.6	-45.3	3.35	3	0.341

Supplementary Table 2: Effect of latitude, longitude, habitat type (deciduous, mixed, evergreen), and species (great or blue tit) on the midpoint (Julian day), duration (days), and delay (days) of temperature windows during which temperature most strongly affects laying date. Significant terms (where 95% confidence intervals do not overlap with 0) are shown in bold. The blue tit is used as the reference category for the species term. Deciduous is used as the reference category for the habitat type term.

**Predictor variable**	**Parameter estimate [95% confidence interval]**
**Midpoint**	
Intercept	-3.26 [-21.31/14.67]
**Latitude**	**1.70 [1.34 / 2.06]**
Longitude	0.09 [-0.13 / 0.31]
**Species (Great tit)**	**4.84 [2.51 / 8.65]**
**Habitat (Evergreen)**	**9.56 [4.86 / 14.56]**
Habitat (Mixed)	-0.68 [-4.80 / 2.88]
**Duration**	
**Intercept**	**56.97 [13.64 / 98.99]**
Latitude	-0.01 [-0.85 / 0.86]
Longitude	-0.26 [-0.79 / 0.25]
Species (Great tit)	0.70 [-6.40 / 7.64]

Predictor variable	Parameter estimate [95% confidence interval]
Habitat (Evergreen)	-7.93 [-19.29 / 3.75]
Habitat (Mixed)	-8.81 [-17.25 / 0.81]
**Delay**	
**Intercept**	**38.62 [21.68 / 56.25]**
Latitude	-0.21 [-0.57 / 0.13]
**Longitude**	**-0.28 [-0.49 / -0.07]**
Species (Great tit)	-2.28 [-5.40 / 0.32]
Habitat (Evergreen)	1.74 [-2.90 / 6.42]
Habitat (Mixed)	1.78 [-1.70 / 5.62]

Supplementary Table 3: Effect of species (great or blue tit), precipitation, and habitat type (deciduous, mixed, evergreen) on the strength of phenological sensitivity (days/°C). Significant terms (where 95% confidence intervals do not overlap with 0) are shown in bold. The blue tit is used as the reference category for the species term and deciduous habitat is the reference category for habitat type.

**Predictor variable**	**Parameter estimate [95% confidence interval]**
**Intercept**	**3.79 [3.58 / 4.05]**
**Habitat (Evergreen)**	**-0.86 [-1.28 / -0.44]**
**Habitat (Mixed)**	**-0.74 [-1.05 / -0.37]**
**Precipitation**	**0.45 [0.35 / 0.72]**
**Species (Great tit)**	**0.29 [0.04 / 0.56]**

Supplementary Table 4: Effect of latitude, longitude, habitat type (deciduous, mixed, evergreen), and species (great or blue tit) on the midpoint (Julian day), duration (days), and delay (days) of temperature windows during which temperature most strongly affects laying date. Models including interactions between species and both latitude and longitude. Significant terms (where 95% confidence intervals do not overlap with 0) are shown in bold. The blue tit is used as the reference category for the species term. Deciduous is used as the reference category for the habitat type term.

**Predictor variable**	**Parameter estimate [95% confidence interval]**
**Midpoint**	
Intercept	5.40 [-27.03 / 23.72]
**Latitude**	**1.49 [1.13 / 2.13]**
**Longitude**	**0.41 [0.11 / 0.69]**
Species (Great tit)	-11.47 [-34.42 / 35.84]
**Habitat (Evergreen)**	**9.58 [5.36 / 13.65]**
Habitat (Mixed)	-0.29 [-3.80 / 2.81]
Species (Great tit):Latitude	0.39 [-0.52 / 0.86]
**Species (Great tit):Longitude**	**-0.52 [-0.88 / -0.11]**
**Duration**	
Intercept	38.52 [-23.84 / 99.31]
Latitude	0.40 [-0.81 / 1.64]

Predictor variable	Parameter estimate [95% confidence interval]
**Longitude**	**-0.67 [-1.40 / -0.002]**
Species (Great tit)	33.66 [-47.67 / 115.05]
Habitat (Evergreen)	-8.02 [-19.02 / 3.34]
Habitat (Mixed)	-9.28 [-17.47 / 0.03]
Species (Great tit):Latitude	-0.74 [-2.35 / 0.87]
Species (Great tit):Longitude	0.71 [-0.17 / 1.60]
**Delay**	
**Intercept**	**41.81 [21.43 / 66.33]**
Latitude	-0.25 [-0.73 / 0.17]
**Longitude**	**-0.50 [-0.79 / -0.27]**
Species (Great tit)	-6.62 [-39.22 / 20.54]
Habitat (Evergreen)	2.04 [-1.86 / 6.08]
Habitat (Mixed)	1.57 [-1.42 / 4.84]
Species (Great tit):Latitude	0.02 [-0.53 / 0.65]
Species (Great tit):Longitude	0.35 [-0.06 / 0.72]

Supplementary Table 5: Effect of species (great or blue tit), precipitation, and habitat type (deciduous, mixed, evergreen) on the strength of phenological sensitivity (days/°C). Models including interactions between species and all other fixed effects terms. Significant terms (where 95% confidence intervals do not overlap with 0) are shown in bold. The blue tit is used as the reference category for the species term and deciduous habitat is the reference category for habitat type.

**Predictor variable**	**Parameter estimate [95% confidence interval]**
**Intercept**	**3.81 [3.61 / 4.05]**
Species (Great tit)	0.18 [-0.11 / 0.46]
**Habitat (Evergreen)**	**-1.09 [-1.63 / -0.55]**
**Habitat (Mixed)**	**-0.79 [-1.19 / -0.35]**
**Precipitation**	**0.57 [0.47 / 0.91]**
Species (Great tit):Habitat (Evergreen)	0.43 [-0.26 / 1.20]
Species (Great tit):Habitat (Mixed)	0.13 [-0.36 / 0.73]
Species (Great tit):Precipitation	-0.08 [-0.43 / 0.28]

Supplementary Table 6: Removal of study sites where temperature data from the E-OBS Gridded Dataset v17.0 is unavailable (Vlieland & Sicily). Effect of latitude, longitude, habitat type (deciduous, mixed, evergreen), and species (great or blue tit) on the midpoint (Julian day), duration (days), and delay (days) of temperature windows during which temperature most strongly affects laying date. Significant terms (where 95% confidence intervals do not overlap with 0) are shown in bold. The blue tit is used as the reference category for the species term. Deciduous is used as the reference category for the habitat type term.

**Predictor variable**	**Parameter estimate [95% confidence interval]**
**Midpoint**	
Intercept	-3.99 [-22.59 / 13.20]
**Latitude**	**1.71 [1.36 / 2.08]**
Longitude	0.10 [-0.14 / 0.30]
**Species (Great tit)**	**5.31 [2.91 / 9.08]**
**Habitat (Evergreen)**	**9.39 [4.81 / 14.59]**
Habitat (Mixed)	-1.46 [-5.68 / 2.35]
**Duration**	
**Intercept**	**56.88 [10.14 / 99.24]**
Latitude	-0.01 [-0.85 / 0.93]
Longitude	-0.27 [-0.86 / 0.22]

Predictor variable	Parameter estimate<br>[95% confidence interval]
Species (Great tit)	0.97 [-6.54 / 8.06]
Habitat (Evergreen)	-7.79 [-19.37 / 4.97]
Habitat (Mixed)	-8.39 [-17.41 / 2.21]
**Delay**	
**Intercept**	**39.42 [21.39 / 56.81]**
Latitude	-0.22 [-0.57 / 0.14]
**Longitude**	**-0.30 [-0.52 / -0.09]**
**Species (Great tit)**	**-2.60 [-5.84 / -0.01]**
Habitat (Evergreen)	2.08 [-2.63 / 7.06]
Habitat (Mixed)	3.01 [-0.78 / 7.05]

Supplementary Table 7: Removal of study sites where temperature data from the E-OBS Gridded Dataset v17.0 is unavailable (Vlieland & Sicily). Effect of species (great or blue tit), precipitation, and habitat type (deciduous, mixed, evergreen) on the strength of phenological sensitivity (days/°C). Significant terms (where 95% confidence intervals do not overlap with 0) are shown in bold. The blue tit is used as the reference category for the species term and deciduous habitat is the reference category for habitat type.

**Predictor variable**	**Parameter estimate [95% confidence interval]**
**Intercept**	**3.92 [3.77 / 4.18]**
**Habitat (Evergreen)**	**-0.84 [-1.22 / -0.47]**
**Habitat (Mixed)**	**-0.66 [-1.00 / -0.28]**
**Precipitation**	**0.56 [0.48 / 0.88]**
**Species (Great tit)**	**0.26 [0.03 / 0.49]**

Supplementary References

1. Haylock, M. R. *et al.* A European daily high-resolution gridded data set of surface temperature and precipitation for 1950–2006. *Journal of Geophysical Research: Atmospheres* **113**, D20119 (2008).
2. Campbell, J. On Temme's algorithm for the modified Bessel function of the third kind. *ACM Transactions on Mathematical Software (TOMS)* **6**, 581–586 (1980).
3. Rousset, F. & Ferdy, J.-B. Testing environmental and genetic effects in the presence of spatial autocorrelation. *Ecography* **37**, 781–790 (2014).
4. van de Pol, M. *et al.* Identifying the best climatic predictors in ecology and evolution. *Methods Ecol. Evol.* **7**, 1246–1257 (2016).
