# Supplementary material for: Bird populations most exposed to climate change are less sensitive to climatic variation
Source: Nat Commun. 2022 Apr 19;13:2112. doi: 10.1038/s41467-022-29635-4 (PMC9018789; doi:10.1038/s41467-022-29635-4)
Supplement: Supplementary file 3 — Description of Additional Supplementary Files [file 41467_2022_29635_MOESM3_ESM.docx]

**Description of Additional Supplementary Files**

**File name:** Supplementary Data 1

**Description:** Location, temperature window characteristics, phenological sensitivity, climate change exposure, and expected phenological advancement for all studied populations. Those populations where P_ΔAICc_ was ≤0.05 and top windows were > 14 days are shaded in grey (47 populations). For all other populations we could not confidently exclude the identified temperature window from the effects of multiple testing. These populations were excluded from further analysis. Estimates of phenological sensitivity, intercepts, and effect of year are determined using a structural equation model that accounts for potential shared trends between temperature and phenology. Note that the units of these coefficients are in negative April days, where days before March 31^st^ are positive and those after March 31^st^ are negative. This was done so that more sensitive populations have a more positive slope. Climate change exposure and expected phenological advancement are only included for those 47 populations where temperature windows could be confidently differentiated from effects of multiple testing. Expected phenological advancement is the product of phenological sensitivity and climate change exposure.
