# Supplementary material for: Bird populations most exposed to climate change are less sensitive to climatic variation
Source: Nat Commun. 2022 Apr 19;13:2112. doi: 10.1038/s41467-022-29635-4 (PMC9018789; doi:10.1038/s41467-022-29635-4)
Supplement: Supplementary file 5 — Reporting Summary [file 41467_2022_29635_MOESM5_ESM.pdf]

nature portfolio

Corresponding author(s): Liam D. Bailey

Last updated by author(s): Feb 9, 2022

Reporting Summary

Nature Portfolio wishes to improve the reproducibility of the work that we publish. This form provides structure for consistency and transparency in reporting. For further information on Nature Portfolio policies, see our Editorial Policies and the Editorial Policy Checklist.

Statistics

For all statistical analyses, confirm that the following items are present in the figure legend, table legend, main text, or Methods section.

n/a	Confirmed	
☐	☒	The exact sample size (n) for each experimental group/condition, given as a discrete number and unit of measurement
☒	☐	A statement on whether measurements were taken from distinct samples or whether the same sample was measured repeatedly
☐	☒	The statistical test(s) used AND whether they are one- or two-sided<br>*Only common tests should be described solely by name; describe more complex techniques in the Methods section.*
☐	☒	A description of all covariates tested
☐	☒	A description of any assumptions or corrections, such as tests of normality and adjustment for multiple comparisons
☐	☒	A full description of the statistical parameters including central tendency (e.g. means) or other basic estimates (e.g. regression coefficient) AND variation (e.g. standard deviation) or associated estimates of uncertainty (e.g. confidence intervals)
☐	☒	For null hypothesis testing, the test statistic (e.g. F, t, r) with confidence intervals, effect sizes, degrees of freedom and P value noted<br>*Give P values as exact values whenever suitable.*
☒	☐	For Bayesian analysis, information on the choice of priors and Markov chain Monte Carlo settings
☐	☒	For hierarchical and complex designs, identification of the appropriate level for tests and full reporting of outcomes
☐	☒	Estimates of effect sizes (e.g. Cohen's d, Pearson's r), indicating how they were calculated

Our web collection on statistics for biologists contains articles on many of the points above.

Software and code

Policy information about availability of computer code

Data collection	No software was used for data collection
Data analysis	Analysis was conducted using R (v4.0.3) in RStudio (v1.3.959). All major packages used in R were available on the Comprehensive R Archive Network (CRAN). Analysis were conducted primarily using the following packages:<br>climwin (v1.2.3)<br>spaMM (v3.9.25)<br>lavaan (v0.6.9)<br><br>All code used for analysis is stored in GitHub repository LiamDBailey/baileyetal2021 and archived on Zenodo (https://doi.org/10.5281/zenodo.6027546).

For manuscripts utilizing custom algorithms or software that are central to the research but not yet described in published literature, software must be made available to editors and reviewers. We strongly encourage code deposition in a community repository (e.g. GitHub). See the Nature Portfolio guidelines for submitting code & software for further information.

Data

Policy information about availability of data

All manuscripts must include a data availability statement. This statement should provide the following information, where applicable:

- Accession codes, unique identifiers, or web links for publicly available datasets
- A description of any restrictions on data availability
- For clinical datasets or third party data, please ensure that the statement adheres to our policy

The phenology data and population characteristics data used in this study and the temperature data used to run sliding window analysis for Sicily and Vlieland are available in the Zenodo repository (https://doi.org/10.5281/zenodo.5747635) 66. The E-OBS Gridded Dataset v17.0 is freely available on request from the European Climate Assessment & Dataset project (ECA&D; https://www.ecad.eu/). The data generated from sliding time window analysis, randomization and fitting of structural equation models are available in the Zenodo repository (https://doi.org/10.5281/zenodo.5747635) 66. A summary of results for each population generated in this study is also provided in the Supplementary Data 1.

Field-specific reporting

Please select the one below that is the best fit for your research. If you are not sure, read the appropriate sections before making your selection.

☐ Life sciences ☐ Behavioural & social sciences ☒ Ecological, evolutionary & environmental sciences

For a reference copy of the document with all sections, see nature.com/documents/nr-reporting-summary-flat.pdf

Ecological, evolutionary & environmental sciences study design

All studies must disclose on these points even when the disclosure is negative.

Study description	We collated phenological data from 67 existing long-term studies of hole-nesting bird populations (great tit and blue tit) across Europe (34 great tit and 33 blue tit; in 27 cases data for both species were collected from the same study site). All populations used had at least 9 years of data, as data of this length has previously been used to successfully to conduct sliding window analyses (https://doi.org/10.1098/rspb.2016.1875). In total, we collated mean annual first egg laying date information in 1,065 years for great tits and 945 years for blue tits. No experimental treatments or interactions between treatments were applied to the data. We used a sliding window approach to quantify phenological senstivity (change in phenology per 1 unit change in temperature [C]) and climate change exposure (change in temperature [C/year]) for each unique populations. For each sliding window analysis we also determined the window midpoint, duration (days), and delay (difference between window midpoint and mean laying date of the population across all years).<br><br>We first analysed the effect of latitude, longitude, species (great tit or blue tit), and habitat type (deciduous, evergreen, mixed) on window characteristics (midpoint, duration, and delay). The habitat type of each population was defined using site descriptions from data owners. Populations with deciduous dominant tree species were defined as 'deciduous' (n = 33), while those with evergreen dominant species were defined as 'evergreen' (n = 7). Populations with both deciduous and evergreen dominant species were considered mixed (n = 27). Two populations (Sagunto, Spain; Avignon, France) were situated in plantations of orange (Citrus x aurantium; evergreen) and apple/pear (Malus domestica and Pyrus spp.; deciduous) respectively. Due to limited sample size, populations in broad-leafed (n = 2) and needle-leafed (n = 5) evergreen habitats were grouped together.<br><br>We next analysed the effect of habitat type (deciduous, evergreen, mixed), species (great tit and blue tit), and annual precipitation patterns on phenological sensitivity, accounting for spatial auto-correlation using a Matern correlation function. Precipitation was derived from the principal component analysis of Metzger et al. (2005; https://doi.org/10.1111/j.1466-822X.2005.00190.x) which incorporates variation in precipitation (mm) across multiple months over the year to provide a quantification of annual precipitation patterns at each study site. All models accounted for crossed design (multiple species studied at the same site) using random intercept for study site. Uncertainty in mean phenology in each population/species/year was accounted for by weighting models with the inverse of standard error. Finally, we estimated the correlation between phenological sensitivity and climate change exposure to understand how intra-specific variation in these two traits may influence a population expected phenological advancement over time (change in phenology/year).
Research sample	Study uses historically collected phenology (laying date) data from populations of Parus major and Cyanistes caeruleus across Europe. Each dataset included estimates of first egg laying date for first clutches, determined by regular nest checks.
Sampling strategy	Phenology data from long-term great tit (Parus major) and blue tit (Cyanistes caeruleus) studies was sampled opportunistically. Beginning in September 2017 co-authors LDB and SJGV collated available phenology data from great tit (Parus major) and blue tit (Cyanistes caeruleus) from across Eurasia and North Africa. LDB and SJGV contacted all research groups known to be working on P. major or C. caeruleus via email. Candidate research groups were identified from authorship in previously published papers and through contacts made at the Hole-Nesting Bird Conference 2017. Data was collated from all interested research groups where at least 9 years of laying date phenology data had been collected at the time of contact (2017). We included only those populations with at least 9 years of data as data of this length has previously been used to successfully to conduct sliding window analyses (https://doi.org/10.1098/rspb.2016.1875). All populations that met these criteria were included. Populations were not excluded for any other reasons.
Data collection	Data were recorded by corresponding data owners at each field site. Data were collated by LDB and SJGV for analysis.

Timing and spatial scale	Contacting research groups and collating phenological datasets began in September, 2017 and ended in December, 2017. In all populations, phenological data was collected annually during the breeding season of P. major and C. caeruleus (exact dates varied with species, latitude, year, and study population). Phenological data on P. major was collected every year starting in 1953 and ending in 2017 (the year when population data were received from co-authors). Phenological data on C. caeruleus was collected every year starting in 1954 and ending in 2017 (the year when population data were received from co-authors). Data were collected from across Europe. Sampled populations ranged latitudinally from 37.6N (Italy) to 69.8N (Finland), with the northern most populations close to the northern range limit of both species. Populations ranged in longitude from -3.99W (UK) to 36.85E (Russia).
Data exclusions	Years of data were excluded if phenology was recorded in fewer than 2 first nests for the season, as in this case standard errors could not be calculated. Populations were excluded if the number of available years of data was less than 9.
Reproducibility	We have no comparable dataset that can be used to repeat the analyses.
Randomization	Our analyses only considered the effects of biotic and abiotic covariates at each study site. There were no experimental treatment groups to which populations could be assigned.
Blinding	We use historically collected phenological data. Data acquisition is therefore necessarily blinded as data were not collected with the intention of being used for this study.

Did the study involve field work? ☐ Yes ☒ No

Reporting for specific materials, systems and methods

We require information from authors about some types of materials, experimental systems and methods used in many studies. Here, indicate whether each material, system or method listed is relevant to your study. If you are not sure if a list item applies to your research, read the appropriate section before selecting a response.

Materials & experimental systems

n/a	Involved in the study
☒	☐ Antibodies
☒	☐ Eukaryotic cell lines
☒	☐ Palaeontology and archaeology
☒	☐ Animals and other organisms
☒	☐ Human research participants
☒	☐ Clinical data
☒	☐ Dual use research of concern

Methods

n/a	Involved in the study
☒	☐ ChIP-seq
☒	☐ Flow cytometry
☒	☐ MRI-based neuroimaging
